# Effects of Curing Temperature on Expansion of Concrete Due to ASR

**DOI:** 10.3390/ma17133140

**Published:** 2024-06-27

**Authors:** Yongfu Yang, Min Deng, Liwu Mo, Wei Li

**Affiliations:** 1 College of Materials Science and Engineering, Nanjing Tech University, Nanjing 210009, Chinaandymoliwu@njtech.edu.cn (L.M.); 2 School of Materials Science and Engineering, Luoyang Institute of Science and Technology, Luoyang 471023, China; 3 State Key Laboratory of Materials-Oriented Chemical Engineering, Nanjing 210009, China

**Keywords:** curing temperature, ASR expansion, pore solution, concentration of OH^−^ ions, activity of NaOH_(aq)_

## Abstract

In the laboratory study of alkali–silica reaction (ASR), models attempt to predict the service life of concrete due to ASR by correlating the performance of concrete at high and low temperatures. However, the consequences of elevating temperature are not so encouraging. In this paper, the influence of temperature on the expansion of 2-graded concrete and 3-graded concrete caused by ASR was investigated by curing the concrete under different temperatures ranging from 40 °C to 80 °C. Increased temperature resulted in rapid expansion at the early stages, but the expansion rate of concrete prisms cured at the higher temperatures (70 °C and 80 °C) was slowed down at the later stages, and concrete prisms cured at 50 °C or 60 °C showed the highest expansions during the experimental period. The chemical analysis results of the pore solution expressed from the concrete show that the ASR expansion is significantly influenced by the [OH^−^]: the decrease in [OH^−^] leads to the retardation of the ASR expansion. The decrease in [OH^−^] is attributed to the consumption of OH^−^ ions for the alkali–silica reaction and the decrease in activity of NaOH(aq) influenced by the temperature. For large cross-section specimens, the OH^−^ within the concrete for alkali–silica reactions cannot be effectively compensated by the external alkali solution. In the accelerated test to evaluate ASR for large cross-section specimens, a curing temperature of less than 60 °C is suggested. This study provides critical insights into the temperature dependency of ASR expansion of concrete, offering a curing temperature range for developing predictive models of ASR expansion under varied environmental conditions.

## 1. Introduction

An alkali–silica reaction (ASR) is a chemical reaction between the reactive silica component contained in aggregate and hydroxyl ions (OH^−^) from the pore solution of concrete [1]. This reaction produces alkali–silicate gel (ASR gel), which can absorb water and cause deleterious expansion, even cracking, in the concrete. Structures can be severely damaged by ASR expansion and are irreparable. It is, therefore, very important to assess the risk of ASR in concrete and to take appropriate measures in advance to prevent ASR expansion. The use of non-reactive aggregates is the most reliable measure to prevent concrete deterioration due to ASR. However, this option is often not practical due to the limited availability of non-reactive aggregates in many locations. In China, alkali-reactive aggregates are widely distributed [2], and their use may be an unavoidable choice, e.g., the dam concretes for the Jinping Hydroelectric Project located in Sichuan Province have no choice but to use alkali-reactive sandstone as a coarse aggregate.

Although ASR is adequately understood, it is still a challenge to assess the risk of deterioration in concrete containing alkali-reactive aggregates. In the laboratory study of ASR, in order to correlate the performance of concrete at high and low temperatures, experimental studies on concrete specimens are typically performed under accelerated conditions by increasing the curing temperature, and some ASR models are proposed to predict the future performance and progression of ASR in structures in service. Although many studies have investigated the influence of temperature on the expansion rate and the final expansion [3,4,5], the relationship between the final expansion and the temperature dependence remains unclear. Tang et al. [6] and Poole et al. [7] presented models based on an Arrhenius equation to extrapolate from laboratory experiments to the field. They found that the measured rate constants of ASR at different temperatures fit the Arrhenius equation well, and the reaction rate at normal temperature could be estimated by extrapolating the regression line through the results at higher temperatures. According to Larive’s work [8], temperature does not affect the final expansion, but it does affect the expansion kinetics [9], and the temperature dependence of the kinetics of ASR expansion follows Arrhenius’ law. The expansion curve at a certain reference temperature has been required to estimate some parameters, and models have been developed by Ulm et al. [10], Farage et al. [11], and Comi et al. [12]. However, Diamond et al. [13] reported that the final expansion was greater at 20 °C than at 40 °C. Kawabata et al. [14] proposed the alkali-wrapped concrete prism test (AW-CPT) to evaluate the effect of temperature on ASR expansion, avoiding the effect of alkali leaching and moisture loss, and the results showed that the final expansion was greater at 40 °C than at 60 °C. Chatterji et al. [15] studied the alkali–silica reaction of two types of sand, and the results showed that for any sand and salt concentration, the expansion increased with the decreasing temperature. Fournier et al. [3] discussed the results of an interlaboratory study on the accelerated concrete prism test (i.e., 60 °C, RH > 95%) for ASR. With increasing temperature, the main expansion phase of the test prisms at 60 °C is often complete after 3 months of testing. Lindgård et al. [16] discussed how exposure conditions influence prism expansion: for prisms exposed to 60 °C, the rate and amount of alkali leaching is the main controlling factor for the prism expansion. Ideker et al. [17] reported that expansions due to alkali–silica reaction (ASR) in the accelerated concrete prism test (ACPT-60 °C) showed a significant reduction at 13 weeks compared to 52 weeks testing in the standard concrete prism test (CPT-38 °C).

Temperature is a critical factor in the alkali–silica reaction, while the expansion pressure exerted by the ASR gel inside the reactive aggregates is a key factor in the expansion process of concrete. Some ASR expansion models describing the expansion behavior associated with the chemical reaction have been developed by Dunant et al. [18], Putatatsananon et al. [19], Multon et al. [20], Takahashi et al. [21], and Miura et al. [22]. However, the present evaluation methods are mainly based on the results of mortar bars or concrete specimens (75 mm × 75 mm × 250 mm, CPT prism), in which the aggregate size is smaller than that of practical concrete. The crack pattern, such as onion skin or sharp cracks [23] in the aggregate, is strongly dependent on the reactive rock type and the random distribution of the expansion sites in the aggregate. The size of the aggregate can strongly affect the alkali transfer pathways and the ASR gel trapped within the aggregate [24]. The results obtained from the mortar bar or CPT prism are not well correlated with the ASR expansion behavior of actual concrete.

The development of accurate and reliable performance tests for the durability of concrete is still a challenge. As stated by Thomas et al. [25], the appropriate benchmark of a laboratory performance test is against real concrete structures or, as a surrogate, against large concrete blocks exposed to natural weathering conditions. However, such field experience is extremely time-consuming. Therefore, an accelerated performance test to evaluate the ASR expansion of concrete is very necessary. According to Hobbs [26] and Glasser [27], the key parameters influencing the rate and extent of alkali–silica reactions are temperature, alkali content, and humidity. In this paper, the 3-graded concrete prism with 300 mm × 300 mm × 500 mm and the 2-graded concrete prism with 150 mm × 150 mm × 550 mm were cast using the practical aggregate, Jingping sandstone. Compared to the CPT prism, the cross-section of samples was enlarged, even for the 3-graded concrete cured in an alkali solution, to avoid alkali leaching. The influence of curing temperature on the ASR expansion of large concrete blocks was studied. It tends to evaluate the effect of temperature on ASR expansion and the temperature dependence of ASR expansion. 

## 2. Materials and Methods

### 2.1. Materials

The coarse aggregate selected for this study was sandstone used in a dam located in Liangshan, Sichuan Province, China. The aggregate shows a mosaic structure consisting of quartz, mica, feldspar, calcite, and chlorite, according to the petrographic analysis. A total of 10% of microcrystalline quartz was considered to be the reactive phase, which is aggregated in the rock. The expansions according to the accelerated mortar bar method (ASTM C1260 [28]) in 14 days and the concrete prism test method (ASTM C1293 [29]) in 1 year are 0.19% and 0.04%, respectively. The coarse aggregate is potentially alkali reactive. The fine aggregate used in the concrete was non-reactive river sand. Portland cement was selected on the basis of its alkali content. Cement LA is a low-alkali cement (0.56% Na_2_O_eq_ by mass) and the chemical composition of the cement is given in Table 1. Polycarboxylate superplasticizer (SP, 1.49% Na_2_O_eq_ by mass) was added to the concrete to ensure good workability. Chemical-grade sodium hydroxide NaOH was used to adjust the alkali content of the concrete.

### 2.2. Sample Preparation and Curing Conditions

The 3-graded concrete and the 2-graded concrete prisms were cast, respectively. The mixes of the laboratory concrete were based on those of the field concretes, but some parameters were adjusted: the alkali content in the concrete was increased to 5.25 kg/m^3^ by adding NaOH to the mixing water, and fly ash was replaced by cement. The water–cement ratio of concrete is 0.5. For the 3-graded concrete, the sand ratio is 39%, and the mass ratio of 5–20 mm aggregate/20–40 mm aggregate/40–80 mm aggregate is 3:3:4. For the 2-graded concrete and the reference concrete, the sand ratio is 30%, and the 5–20 mm aggregate/20–40 mm aggregate is 5:5 by mass. For the reference concrete, the alkali content was 1.60 kg/m^3^, and alkali was supplied only by the cement and superplasticizer. The mixing proportions of the concretes are given in Table 2. Polycarboxylate superplasticizer was added to the fresh concretes to ensure they had the same slump.

**Figure 1:**
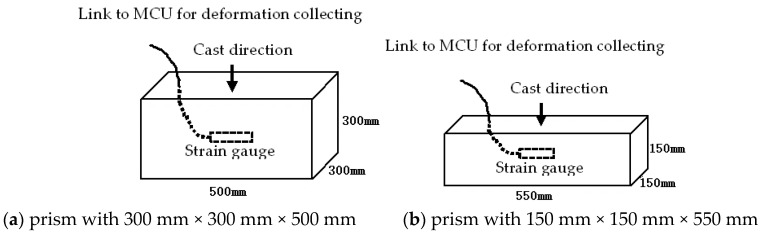
Schematic of stain gauge embedded in sample.

The 3-graded concrete prisms with a size of 300 mm × 300 mm × 500 mm, the 2-graded concrete prisms with a size of 150 mm × 150 mm × 550 mm, and the reference concrete prisms with a size of 150 mm × 150 mm × 550 mm were cast. A strain gauge was embedded in the concrete (Figure 1) to measure ASR expansion. In addition, 12 prisms of the 3-graded concrete with dimensions of 300 mm × 300 mm × 500 mm and 18 cubes of the 2-graded concrete with dimensions of 150 mm × 150 mm × 150 mm were cast for pore solution extraction at a specific period. Samples were cured for 5 days at 22 ± 1 °C and RH ≥ 95% and then stored in the curing boxes. Prisms of the 3-graded concrete were immersed in 0.7 mol/L NaOH solution. Prisms or cubes of the 2-graded concrete were stored above water, i.e., cured in the moisture. All concrete prisms were stored vertically in the curing boxes, and the curing temperature was set at 40, 50, 60, 70, and 80 °C, respectively. 

### 2.3. Expansion Measurement

Strain gauges embedded in the concrete were used to measure the deformation due to ASR expansion. The sensors were linked to the microcontroller unit (MCU) system, and deformation data was collected at 12 h intervals. 

### 2.4. Internal Moisture Content and Open Porosity of Concrete

The evaporable water content was determined to represent the internal humidity of the concrete, and the open porosity of the concrete was calculated. Concrete cubes with sizes of 100 mm × 100 mm × 100 mm were cast and cured in moisture at 40 °C, 60 °C, and 80 °C, respectively. After a long-term (1 year) saturation by the moisture in the curing boxes, the mass of the specimens was measured, then they were finally dried at 105 °C and weighed. The internal moisture content (*C_m_*) and the concrete open porosity (p) are calculated using the following equations:

(1) $$C_{m}=\frac{m_{0}-m_{i}}{m_{i}}\times 100\%$$

(2) $$p=\frac{V_{water}}{V_{concrete}}=\frac{(m_{0}-m_{i})\cdot \rho_{concrete}}{m_{0}\cdot \rho_{water}}\times 100\%$$

where $V_{water}$ is the pore volume in concrete, $V_{concrete}$ is the volume of concrete, $m_{0}$ is the mass of the moisture-saturated specimen, $m_{i}$ is the mass of the dry specimen, $\rho_{concrete}$ is the density of concrete, and $\rho_{water}$ is the density of water.

### 2.5. Ion Concentration of Pore Solution

The concretes for pore solution were cured at 40, 60, and 80 °C, respectively. The 3-graded concrete prisms were immersed in 0.7 mol/L NaOH solution, while the 2-graded concrete cubes were cured in moisture. At certain ages, the power supply to the curing chambers was deactivated, allowing the containers to cool to ambient temperature. Cores with the diameter of 160 mm and the length of 200 mm (Φ160 mm × 200 mm) were taken out from the 3-graded concrete and divided into two parts: the inner and the outer with the size of Φ160 mm × 100 mm. Samples for pore solution were crushed and then subjected to high-pressure expression (1200 MPa), following the method outlined by Barneyback [30]. Following expression, approximately 200 μL of pore solution (with an accuracy of 5 μL) was transferred to a 50 mL volumetric flask and diluted with deionized water to reach volume. The concentration of Na and K were determined using a flame photometer. The concentration of OH^−^ ions was assessed through direct titration with 0.01 mol/L HCl (with an accuracy of 0.0001 mol/L) using the phenolphthalein endpoint. Chemical analysis was carried out at 22 ± 2 °C. In the experimental process, some measures were taken to keep the pore solution from carbonating, such as fresh deionized water was always used. 

## 3. Results and Discussion

### 3.1. Deformation of the Reference Concrete

**Figure 2:**
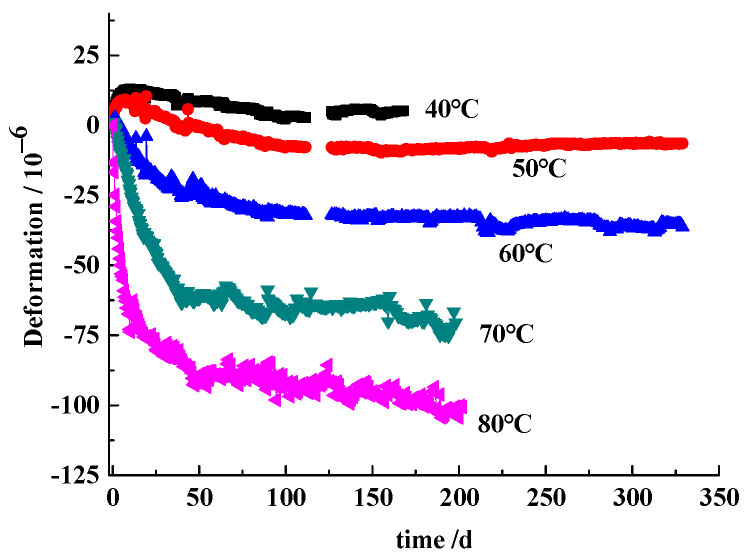
Deformation of the reference concrete stored over water.

The deformation of the concretes includes the expansion due to ASR and the shrinkage due to the cement hydration. Figure 2 presents the deformation at different curing temperatures obtained on the reference concrete. It is evident that the concrete experiences shrinkage and this shrinkage becomes more pronounced with higher temperatures. The rapid hydration of cement at elevated temperatures leads to increased shrinkage, particularly during the early age. The reference concrete with low alkali content (1.60 kg/m^3^ Na_2_O_eq_) is regarded as non-reactive concrete, though they were cast by reactive aggregate. 

### 3.2. ASR Expansion

**Figure 3:**
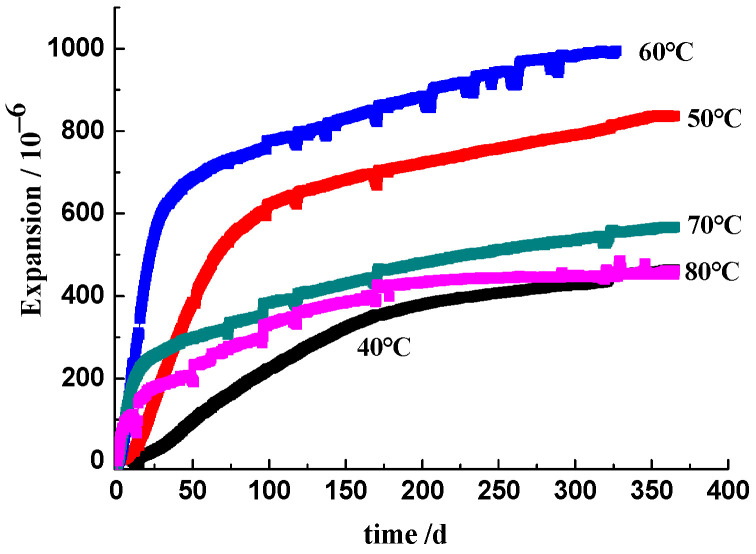
Deformation of the 3-graded concrete immersed in 0.7 mol/L NaOH solution.

**Figure 4:**
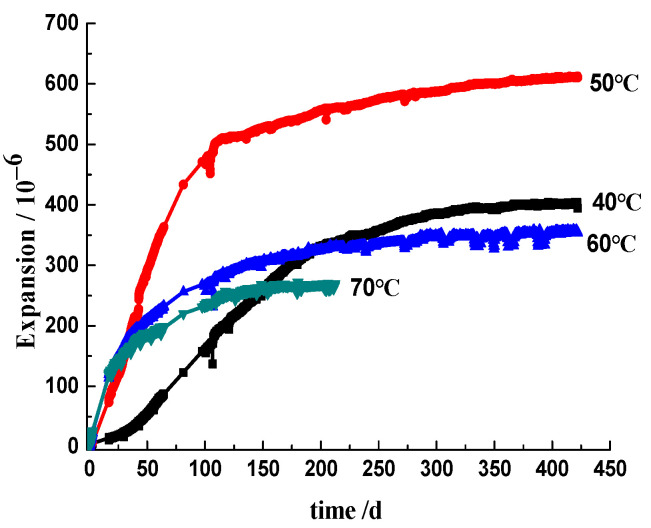
Deformation of the 2-graded concrete stored over water.

Figure 3 and Figure 4 show the deformation at different curing temperatures obtained on the 3-graded concrete and the 2-graded concrete, respectively. The induction period of the expansion depends on the curing temperature. At a low temperature (40 °C), the curves show an S-shape, indicating a very slow expansion rate at first, then a rapid increase, and finally, a gradual slowing down. As the temperature increased, the specimens expanded rapidly after a short latent period, followed by a low rate of expansion. For the 3-graded concrete prisms immersed in alkali solution, the expansions in 1 year under five curing temperatures of 40, 50, 60, 70, and 80 °C are 0.050%, 0.084%, 0.099%, 0.057%, and 0.046%, respectively. The prism cured at 60 °C produced the highest expansion during the experimental period. For the 2-graded concrete prism cured in moisture at 70 °C, the curve indicates a very rapid ASR expansion rate in the first 25 days, then a gradual slowing down in the period of 25–150 days. After 150 days, the expansion remains almost unchanged, and the expansion up to a final asymptote is 0.027%. For the 2-graded concrete prism cured at 60 °C, it expands rapidly in the first 40 days, then a gradual slowing down in the period of 40–250 days. The specimen remains stable after 250 days, with an expansion of 0.035% at 420 days. For the prism cured at 50 °C, the expansion rate remains relatively fast before 100 days, then slows down. The expansion at 420 days is 0.061%. For the prism cured at 40 °C, after a latent period of about 25 days, the specimen begins to expand with the slowest expansion rate. However, in the later age, its expansion exceeds that of specimens cured at 60 °C and 70 °C. At 420 days, the expansion is 0.040%. The prism cured at 50 °C in moisture shows the highest expansion. The expansion of the 2-graded concrete prism cured in moisture is always lower than that of the 3-graded concrete prisms immersed in alkali solution at the same curing temperature. Whether the concrete is cured in moisture or alkaline solution, within the curing temperature range of 40–80 °C, the higher the curing temperature, the faster the initial ASR expansion of concrete prisms. Increasing the curing temperature significantly accelerates the ASR expansion process of concrete. However, increasing the curing temperature will also shorten the duration of the rapid expansion stage of concrete, and the earlier the ASR expansion of concrete tends to stabilize. The prisms cured at lower temperatures have a slower initial expansion rate, but they maintain stable growth in the later ages. The expansion rate of concrete prisms cured at the higher temperatures (70 °C and 80 °C) was slowed down at the later ages, and concrete prisms cured at 50 °C or 60 °C showed the highest expansions during the experimental period. ASR expansion of prism cured at the higher temperature is not necessarily larger.

### 3.3. Internal Moisture Content and Porosity

The internal moisture content and the open porosity of concrete samples cured at 40 °C, 60 °C, and 80 °C were tested, and the results are shown in Table 3. The internal moisture content within concrete increases with increasing curing temperature, indicating that concrete samples can maintain a high RH level even at an elevated temperature to ensure that ASR expansion proceeds [16]. The open porosity of the concrete also increased with increasing temperature.

### 3.4. Alkali Content in Pore Solution

Table 4 shows the ion concentrations of the pore solution in the 2-graded concrete cured in moisture. Table 5 shows the ion concentrations of the pore solution in the 3-graded concrete cured in 0.7 mol/L NaOH solution. For each storage temperature, the [Na^+^], [K^+^], and [OH^−^] of the pore solution in concretes, whether cured in moisture or in alkali solution, tend to decrease with the development of ASR for up to 1 year, except at 40 °C in 7 days, where an increase in the [Na^+^] and [K^+^] is observed. Moreover, the ion concentrations in the pore solution decreased more rapidly as the curing temperature increased. Compared to the concrete cured in moisture, the ion concentrations of concrete cured in alkali solution decreased more slowly. For concrete cured in moisture at 60 °C and 80 °C for 1 year, the [OH^−^] of the pore solution is too low to be detected. For concrete cured in alkali solution, the [K^+^] in the pore solution of the inner concrete at any curing temperature is always higher than that of the outer concrete. The [OH^−^] in the pore solution of the inner concrete cured at 40 °C, 60 °C, and 80 °C for 28 days and at 40 °C for 60 days is higher than that of the outer concrete, but it inverses for concrete cured for 60 days at 60 °C, 80 °C, and for 1 year at each curing temperature. Similar results to the [Na^+^] are also observed in Table 5.

### 3.5. Discussion

The results described in this paper show that the expansion of concrete either cured in moisture or cured in alkali solution and the [OH^−^] in concrete pore solution are greatly influenced by temperature. In general, increasing the curing temperature from 40 °C to 80 °C can accelerate the rate of ASR expansion at early ages. However, in this experiment, the higher temperature of 80 °C did not result in higher final expansion. The results are different from those of Liu [31] and Ke [32]. In their experiments, mortar bars were cured in 1 mol/L NaOH solution, and the results showed that the ASR expansion increased with increasing temperature, i.e., the mortar bar cured at 80 °C showed the highest expansion in the experimental periods. 

#### 3.5.1. Contradiction to the Alkali Leaching

As mentioned in the Introduction, the reduction in the final expansion of concrete stored over water at elevated temperatures has been attributed to the alkali leaching. Many researchers explained that alkali leaching would reduce the alkalinity of the pore solution in the concrete, thus significantly reducing the final expansion [16,20,33]. In this experiment, alkali leaching is inevitable in the 2-graded concrete stored over water, though some measures were taken to avoid or reduce the leaching of alkali from the concrete, such as increasing the cross-section of the concrete prism, prolonging the pre-storage period at room temperature. It cannot be denied that alkali leaching made an important contribution to the reduction in ASR expansion of concrete cured at an elevated temperature in moisture, even when immersed in water. However, for the 3-graded concrete immersed in 0.7 mol/L NaOH solution, the prisms were surrounded by the alkali ions, and alkali leaching from the concrete to the simulated solution was negligible. In addition, the concentration of NaOH solution decreased to 0.6 mol/L after 1 year at 80 °C, indicating that the alkali had penetrated into the concrete to compensate for alkali consumption. At this storage condition, which is similar to that of Ke and Liu’s experiments, the prism cured at 60 °C showed the highest expansion, unlike Ke and Liu’s results: the higher the curing temperature, the higher the expansion obtained. This phenomenon, which occurred in the 3-graded concrete, cannot be explained by alkali leaching.

#### 3.5.2. The [OH^−^] Influenced by Temperature 

The OH^−^ ions in the pore solution were produced by the hydration of the cement and the dissolution of NaOH in the mixing water. Lothenbach et al. [34] investigated the change in concentration of OH^−^ ions in Portland cement paste cured at temperatures ranging from 5 °C to 50 °C. Their results showed that the [OH^−^] increased with increasing temperature and the evolution of hydration. Durand et al. [35] showed a similar result that the [OH^−^] of the pore solution in Portland cement paste cured at 38 °C was maintained at a high level at later ages. It can be concluded that the [OH^−^] will not vary dramatically in concrete if no OH^−^ ions are consumed. In our experiments, comparing Figure 4 and Table 4, the 2-graded concrete prisms cured in moisture expanded rapidly at the early ages with the decline in [OH^−^] in concrete pore solution, followed by a decrease in the rate until a long-term asymptote was reached, accompanying the [OH^−^] decreased to a very low level. Comparing Figure 3 and Table 5, for the 3-graded concrete, prisms expanded continuously after the rapid expansion at the early ages. Simultaneously, the [OH^−^] decreased due to the consumption of OH^−^ ions in concrete, though the concrete prisms were immersed in alkali solution. For concrete prism cured at 80 °C for 1 year, the concentration of OH^−^ ions in the inner concrete decreased to a very low level of 0.029 mol/L, far from the threshold [OH^−^] of 0.25 mol/L suggested by Diamond [36]. In our laboratory, the dissolution kinetics of reactive silica in Jingping sandstone exposed to NaOH solution was studied, and the experimental results indicate that ASR behaves as a first-order reaction, accompanied by the exponential decrease in the [OH^−^] with time [37]. The alkali content and the concentration of OH^−^ in the concrete pore solution play a major role in the development of ASR, especially the role of [OH^−^] in the evolution of ASR [9]. Therefore, the change in alkali content and OH^−^ concentration in the pore solution can well interpret the rate and extent of ASR during laboratory performance testing. The alkali and OH^−^ ions permeate into sandstone aggregates and react with the microcrystal silica phase, producing expansive ASR gels. At the early ages, the alkali–silica reaction proceeds rapidly for the high [OH^−^], and the reaction rate increases with the increasing temperature. Accordingly, the concrete prisms expand rapidly. As the alkali–silica reaction proceeds, OH^−^ ions are continuously consumed, resulting in a decrease in [OH^−^]. When the [OH^−^] decreases to the threshold for initiating ASR expansion, the reaction becomes very slow, and no apparent expansion is observed. For the mortar bar immersed in the alkali solution, ASR expansion of the specimen with small scale section occurred in an alkali-supply environment, but for the prism in 300 mm × 300 mm × 500 mm cured in 0.7 mol/L NaOH solution, the alkali ions in pore solution cannot be compensated effectively from the outside alkali solution.

#### 3.5.3. The Effective [OH^−^] Influenced by Temperature

**Figure 5:**
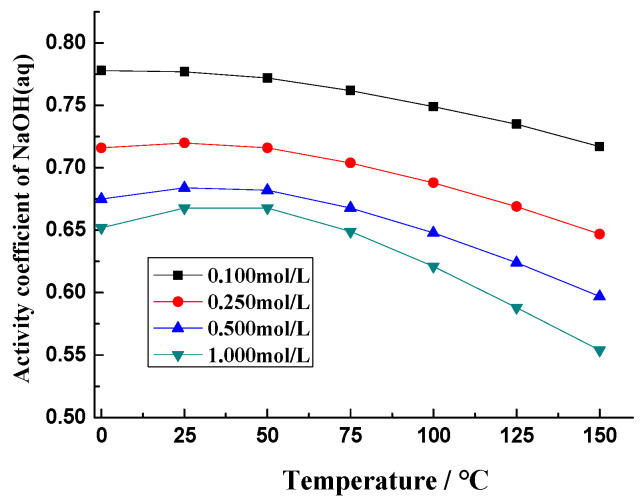
Activity coefficients of NaOH_(aq)_ [38].

The effective [OH^−^] in the pore solution of concrete is also influenced by the curing temperature. NaOH in concrete pore solution will dissociate into Na^+^ and OH^−^, as NaOH_(aq)_ is a strong electrolyte. The degree of ionization depends on the concentration and temperature of the solution. According to the results of Pabalan et al. [38], activity coefficients of NaOH_(aq)_ at 1 bar or saturation pressure are shown in Figure 5. When the concentration of sodium hydroxide is greater than 0.25 mol/L, the activity coefficient increased slightly with the temperature increase from 0 °C to 50 °C, then it declined gradually. When the concentration is less than 0.25 mol/L, the activity coefficient decreases with the increasing temperature. This means that the amount of effective OH^−^ ions in the pore solution of concrete cured at a lower temperature (40 °C) is greater than that of concrete cured at a higher temperature (80 °C) if the concrete has the same content of NaOH. 

**Figure 6:**
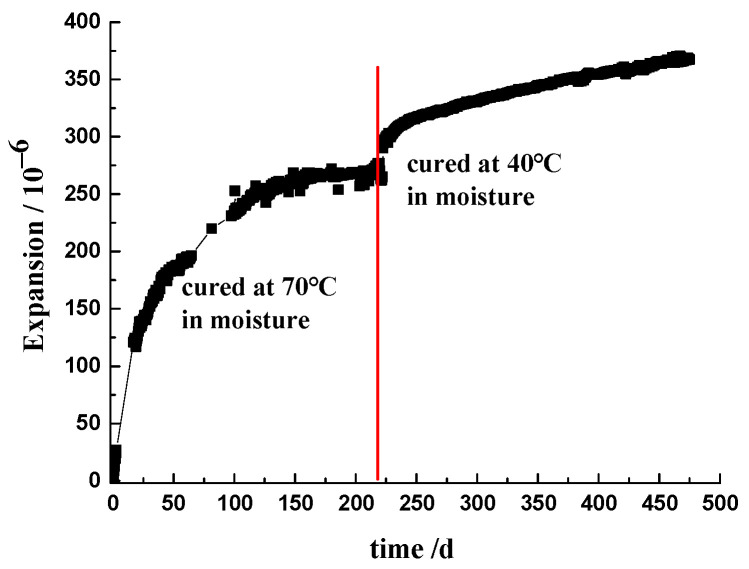
Re-expansion of the prism when transferred from 70 °C to 40 °C of curing condition.

The effective OH^−^ ions play a major role in the ASR expansion of concrete. When the specimen with the 2-graded aggregate cured at 70 °C in moisture for 220 days (the expansion had reached a long-term asymptote) was transferred to the conditions of 40 °C, it re-expanded rapidly and continuously (Figure 6). As mentioned in Table 4, moisture content in concrete decreased with the decrease in temperature, so the re-expansion of the concrete prism is not attributed to the wet swelling of concrete. Notably, samples cured at a high temperature show a high porosity, which means that the re-expansion needs more ASR gel to fill the confined space compared to the prism cured at 40 °C all the time [39]. With the temperature decreased, the amount of effective OH^−^ ions increased, and ASR proceeded again, leading to the re-expansion of the concrete prism.

#### 3.5.4. Temperature Dependency of ASR Expansion

The ASR expansion of concrete is influenced by the effective alkaline ion concentration of the concrete pore solution. At the initial curing stage of concrete, the concentration of alkali ions in the pore solution is high, and the alkali–silica reaction rate is rapid, leading to more ASR gel generation and prisms expanding rapidly at various temperatures. As the curing age prolongs, the concentrations of K^+^, Na^+^, and OH^−^ ions continue to decrease, and the rate of alkali–silicate reaction slows down; as a result, the expansion of the prism cured at various temperatures also slows down. 

The influence of elevating temperature on ASR expansion may manifest in the following ways: (1) When the alkali content in the concrete pore solution is adequate, elevating temperature will accelerate the process of alkali–silica reaction, and ASR gels are mostly confined in a limited space within the aggregate, which induces the rapid ASR expansion, i.e., in the early ages, the higher curing temperature, the greater ASR expansion; (2) As the temperature increases, the [OH^−^] in the concrete pore solution decreases faster to the threshold for initiating ASR expansion, which slows down the rate of ASR expansion; (3) The activity coefficients of OH^−^ ion may decrease with the increasing temperature, which means that the amount of effective OH^−^ ions in pore solution of concrete cured at a higher temperature is less than a lower temperature if the concrete has the same content of NaOH, and this may decrease ASR expansion; (4) Elevating temperature boosts the exudation of ASR gels from the sandstone into the pore solution, which reduces ASR expansion [5,37]. Thus, ASR expansion is comprehensively affected by the curing temperature.

## 4. Conclusions

This study investigated the influence of curing temperature on the ASR expansion of concrete containing reactive sandstone as the coarse aggregate. Two sets of tests were carried out, namely, (1) 3-graded concrete with 300 mm × 300 mm × 500 mm cured in alkali solution and (2) 2-graded concrete with 150 mm × 150 mm × 550 mm cured in moisture. The following conclusions are obtained:

- ASR expansion of concrete shows a strong temperature dependency. Increasing the curing temperature accelerates the expansion rate of ASR in the early ages, which exhibits a positive temperature dependency, while increasing the temperature also accelerates the [OH^−^] reduction, decreases the effective [OH^−^], and boosts the exudation of ASR gels from aggregates, which shows a reduced, negative temperature dependency.
- The ASR expansion is significantly influenced by the [OH^−^] in the pore solution of concrete. Increasing the curing temperature may accelerate the decrease in [OH^−^] and shorten the duration of the rapid expansion stage of concrete. Specimens cured at 50 °C or 60 °C showed the highest expansion during the experimental period. The ASR expansion of specimens cured at higher temperatures was not necessarily greater.
- For the 300 mm × 300 mm × 500 mm specimens, the OH^−^ within concrete for ASR cannot be compensated effectively from the outside alkali solution.
- The temperature dependency of ASR expansion in the large cross-section concrete prisms should be considered when correlating concrete performance at high and low temperatures. In the accelerated test to evaluate ASR for large cross-section specimens, a curing temperature of less than 60 °C is suggested.

## Figures and Tables

**Table 1:** Chemical composition of the cement.

Sample	Chemical Composition/wt.%									
	SiO_2_	Al_2_O_3_	Fe_2_O_3_	CaO	MgO	SO_3_	K_2_O	Na_2_O	L.O.I	Total
Cement LA	19.56	4.28	4.17	63.61	1.27	2.05	0.53	0.21	3.19	98.87

**Table 2:** Mix proportions of the concretes.

Concrete Type	Na_2_O_e_ /(kg/m^3^)	Mix Proportions/(kg/m^3^)						
		Cement	Water	Fine Aggregate	Coarse Aggregate			SP
					5–20 mm	20–40 mm	40–80 mm	
3-graded	5.25	204	102	605	468	468	624	1.8
2-graded	5.25	280	140	614	715	715	-	1.8
Reference	1.60	280	140	614	715	715	-	1.1

**Table 3:** Internal moisture content and porosity of the 2-graded concrete cured in moisture.

Samples Cured at	Internal Moisture Content/%	Porosity/%
40 °C	6.41	7.81
60 °C	6.47	9.30
80 °C	6.55	9.72

**Table 4:** Ion concentration of the pore solution in concrete cured in moisture.

Curing Time/Days		0	7	14	28	180	365
[K^+^]/(mmol/L)	40 °C	212.3	231.6	193.0	155.7	130.7	39.8
	60 °C		141.5	111.2	102.5	70.8	26.5
	80 °C		128.9	106.2	56.6	25.0	23.6
[Na^+^]/(mmol/L)	40 °C	523.5	616.0	557.4	451.8	372.6	262.2
	60 °C		443.7	353.4	300.4	179.3	201.7
	80 °C		403.4	419.5	236.6	94.9	143.4
([K^+^] + [Na^+^])/(mmol/L)	40 °C	735.8	847.6	750.4	607.5	503.3	302.0
	60 °C		585.2	464.6	402.9	250.1	228.2
	80 °C		532.3	525.7	293.2	119.9	167.0
[OH^−^]/(mmol/L)	40 °C	515.8	496.2	441.8	394.9	346.8	220.3
	60 °C		375.3	336.0	233.6	161.4	-
	80 °C		302.3	293.6	112.9	78.2	-

0 d: the sample for pore solution cured at 22 ± 2 °C, RH 100% for 1 day. -: the OH^−^ concentration is too low to be detected.

**Table 5:** Ion concentration of the pore solution in concrete cured in 0.7 mol/L NaOH solution.

Curing Time/Days		Ion Concentration of Pore Solution/(mmol/L)								
		40 °C			60 °C			80 °C		
		[K^+^]	[Na^+^]	[OH^−^]	[K^+^]	[Na^+^]	[OH^−^]	[K^+^]	[Na^+^]	[OH^−^]
0		331.5	786.7	658.4	331.5	786.7	658.4	331.5	786.7	658.4
28	Outer	212.3	676.6	484.0	252.5	558.2	379.4	185.1	455.1	285.4
	Inner	297.3	748.6	503.4	261.0	564.7	453.8	245.4	530.6	301.2
60	Outer	225.6	595.0	378.1	177.9	505.8	287.8	109.2	451.8	165.9
	Inner	253.2	608.1	409.5	160.7	427.3	268.2	148.6	441.0	153.3
365	Outer	88.9	630.4	348.9	56.3	412.8	213.9	26.5	363.0	60.5
	Inner	100.3	546.8	322.7	70.2	402.7	179.4	45.0	388.9	29.3

0 d: the sample for pore solution cured at 22 ± 2 °C, 100% RH for 1 day.

## Data Availability

Data are contained within the article.
